# Distinguishing the Need to Belong and Sense of Belongingness: The Relation between Need to Belong and Personal Appraisals under Two Different Belongingness–Conditions

**DOI:** 10.3390/ejihpe13020025

**Published:** 2023-02-01

**Authors:** Saga Pardede, Velibor Bobo Kovač

**Affiliations:** 1 Department of Psychosocial Health, University of Agder, 4879 Grimstad, Norway; 2 Department of Education, University of Agder, 4604 Kristiansand, Norway

**Keywords:** belongingness-conditions, need to belong, appraisals, social exclusion, social-worthiness, social-value representation, emotional self-expression, social relations

## Abstract

People are frequently caught in the hold between the need to belong and the fear of exclusion. However, these needs might be expressed differently under different belongingness conditions, where other powerful social processes are accentuated. Thus, the need to belong and social exclusion are concepts that are subjectively appraised based on one’s social relations. The present study aims to examine the relationship between the need to belong and five personal appraisals under two different belongingness conditions: (1) social-emotion support and (2) social-value representation. A total of 201 participants from two different groups were presented with 69 different items measuring five personal appraisals (exclusion, shame, social-worthiness, emotional self-expression, and prosocial-relating behaviour). Condition 2, social-value representation with social worthiness being appraised, offered the strongest connection as a significant predictor amongst all appraisals in both conditions, despite both exclusion and shame being indicated as significant predictors, to begin with. Thus, highlighting the appraisal of social worthiness in support of one’s need to socially represent oneself by not being compared to others while being valued as an alternative motive for realising a sense of belongingness. The empirical and theoretical limitations and implications are also discussed.

## 1. Introduction

People have a fundamental need for a psychological and emotional feeling to belong to someone and something that they hold dear [1,2]. There are solid empirical and theoretical grounds to claim that the need to form positive social connections and relatedness is universal and fundamental (e.g., [3,4,5]). The need to belong is based on a motivational human need to maintain interpersonal relationships and positive social bonds, and as such, it becomes significant for our overall development and well-being [6,7]. The need to belong is so potent that some people paradoxically prefer to be in a group of strangers than to be alone [2,8], perhaps because even social acceptance from strangers holds a positive psychological effect, contrary to the painful feeling of being socially excluded [9]. For example, studies show those who experienced unexpected exclusion demonstrated a more negative relational effect in comparison to those who expected a threat to their social connections [10,11,12]. Hence, people experience a severely negative psychological and physical outcome when the need for connection is threatened by potential exclusion e.g., [13,14,15] forging humans to consequently be sensitive to any signs that signal a threat to one’s social bond [15,16].

This suggests that the need to belong can be in many ways contrasted with the fear of social exclusion. Akin to this painful physical experience of not belonging or feeling accepted is the fear of being socially excluded [15]. The fear of social exclusion may present itself with many faces, e.g., rejection, ostracism, and social isolation (e.g., [17,18]) and for a myriad of reasons. Past research has highlighted that people tend to react differently to social exclusion [15,19,20]. Moreover, the more people feel excluded over time, the more lingering their fear of exclusion is at risk [21]. Research shows that social exclusion is associated with a range of negative psychological outcomes such as people acting more impulsively [22] and struggling with loneliness [23]. In connection with this, studies also show that a series of negative emotions arise when people are excluded from groups or social bonds, for example, shame, anger, and sadness (e.g., [24,25,26,27]). Furthermore, research has also demonstrated that people who are rejected can even behave maladaptively towards those who are innocent as opposed to those who are excluding them (for review see, [28]).

In concrete, life situations, it is easy to imagine that people might be caught in the hold between these two powerful processes, namely the need to belong and the fear of exclusion. The one potential end-result of this “hold” is the emergence of one’s prevalent balance of sense of belongingness, i.e., the overall experience that people feel through a sense of acceptance. Throughout the decades, there have been several ways of defining belongingness. In terms of wider definitional understanding, belongingness is related to various types of experiences, for example, the experience of social acceptance [8], social or peer support (e.g., [29,30]), being able to self-identify [31,32] and self-categorisation within group-membership [33]. Thus, there exists a rich body of literature showing that people devote considerable time and energy to forming and maintaining social bonds based on the motivating factor of a sense of belongingness [2,34]. This is expected, considering that the experience of belongingness generates a sense of attachment security and a sense of identity which are significant and necessary for an individual’s overall development [2,6].

On the individual level, the experience of belongingness signifies a personal connection and experience with the world [35]. One’s sense of belongingness can be motivated by a need and concern to connect in a deep emotional meaning, or the concern for a successful self and social representation of oneself [33,36,37], consequently averting the negative psychological outcomes of social exclusion such as impulsiveness [22] and loneliness [23]. It follows that people are drawn to a sense of belongingness on the grounds that belongingness is the fabric that connects the self with others, places, and objects [38,39]. This implies that one’s sense of belongingness, in contrast to the fundamental need to belong, is inherently contextual, and emerges as the result of the individual’s social, cultural, and emotional state within a specific environment, or at the time of specific experience. Considering that the sense of belongingness is based on personal experiences and involvement with social environments [2,40], it is possible to argue that a sense of belongingness is essentially a response to our emotional appraisals of our relations and interactions with others in the given conditions (e.g., [41,42]).

Therefore, a sense of belongingness is linked to an appraisal of one’s concern in social relations and society [18,43,44], or could be perceived as a response to our emotional appraisals of our relations and interactions with others in the given conditions (e.g., [41,42]). For example, when people rely heavily on social approval for a sense of self-worth, shame might become a core driver that can often make people feel as if they do not belong based on this painful appraisal of flawed connection to others and reference to social norms [45]. As Hagerty et al. [39]. described, “*a sense of belongingness occurs when people feel like they are an integral part of a system or environment*” (p. 173). Similarly, Simonsen [46] defines a parallel process as the need for “*identifying with and feeling attachment to social group*” (p. 120).

However, despite the general agreement that the need to belong is a fundamental human need that all people pursue (e.g., [47,48]), there is still less agreement on the construct itself, and more importantly its conceptual relation to the sense of belongingness that emerges under different conditions. In other words, although it is relatively clear that all people *have the need to belong*, it is nevertheless reasonable to assume that this need could be expressed differently under different conditions, where other powerful social processes are accentuated. The common starting point for both sense of belongingness and social exclusion is that both concepts are subjectively appraised based on one’s social relations, holding a strong bearing on one’s motivation and functioning [49]). For example, previous theory (e.g., [26,44]) and empirical research (e.g., [39,50]) indicate that a key to understanding psychological outcomes based on the experience of social exclusion is centred around the appraisal of one’s interpersonal social relations within an environment. This argument follows appraisal theories [41,51,52,53,54] suggesting that emotions are stimulated when individuals evaluate a condition central to their concerns. As such, when the feeling of social exclusion reflects one’s psychological experience, a negative evaluation of one’s sense of belongingness might emerge. It follows that the appraisal of exclusion informs a crisis and causes unpleasant feelings that lead to emotional distress within the human psyche (e.g., [55,56]).

## 2. The Present Study

Based on the above-described theoretical reasoning, the present study aims to examine the relationship between the need to belong and five personal appraisals under two different belongingness-conditions. The first belongingness-condition is termed “social-emotion support” referring to situations where people experience support when they are allowed to be themselves and express their true emotions. The second belongingness-condition is termed the “social-value representation” referring to situations where people experience that it is important to be valued and not compared to others.

Individual appraisals refer to basic psychological experiences that are, on theoretical grounds, expected to be related to the need to belong. The appraisals that are included in the present study are appraisals of exclusion, shame, social-worthiness, emotional self-expression, and prosocial-relating behaviour. The appraisals of exclusion and shame have been chosen due to their previously suggested inverse connection to a sense of belongingness [57,58,59]. For example, the feeling of shame occurs typically when there is a negative appraisal of one’s self-image or social representation based on an awareness of how others or another “judges” the “self” [60]. Similarly, fear of exclusion as an appraisal was chosen due to the varied and multiple negative social experiences that can be activated when a person is rejected or isolated [18,61]. The rationale for including social-worthiness is related to people’s appraisals of worth based on social (dis)approval in concrete situations. It follows that the sense of personal worthiness is, on the theoretical level, expected to be related to the core sense of shame [45]. Thus, people are inherently motivated to maintain a positive image of themselves which is based on perceived social representation and interaction with other people [62]. “Others” represent a powerful source of influence on our self-appraisals, activating potentially internalising processes that might have an impact on one’s basic need to belong [63]. The rationale for including emotional self-expression is based on the general relation between the need to belong and the basic human need for receiving emotional support lasting from infancy to maturity [64]. Thus, connectedness with others is built throughout the stages in the early years based on the ability to reciprocally share emotions and connections [37]. The experience of being accepted in social conditions provides closeness and intimacy with others and these experiences help to reduce situational ambiguity [65]. Finally, the rationale for including prosocial-relating behaviour is based on the strong support across the discipline of motivation and behaviour, where the association between positive social bonds fosters empathic development and capacities between self and others [66,67]. This cognitive framework of oneself in relation to others and the social environment evidently becomes one’s positive internal working model towards an empathic action. Thus, conversely, more empathic individuals are more likely to share their resources. For example, various studies have found how prosocial activities correlate with social acceptance (e.g., [68,69]). As such, people are more likely to act prosocially for the benefits that a sense of belongingness has to offer. Moreover, parallel to this, prosocial action helps as a means of connecting with others [70].

## 3. Method

### 3.1. Procedures and Participants

A total of 201 participants were recruited online across the globe and an online survey using “Google Forms” (an administrative software platform) was conducted. Participants were invited to fill in a demographic section (i.e., gender, age, nationality, and native language), followed by a section where they were asked to respond to a brief reflective section relating to their experiences of one of the two belongingness-conditions. A random allocation tool “Allocate Monster” was used to randomly distribute each participant to one of the two belongingness conditions (“social-emotional support” and “social-value representation”) to avoid overlap in the same pool of participants. Following this, participants responded to a standardised questionnaire containing the main instruments in the study. There were no missing data, as the survey was designed in such a way that each section and item required a response before moving to the next one and section. Participants were informed that their involvement was completely voluntary and that all information would be treated confidentially.

*Participants in condition 1 (social-emotion support):* 107 participants volunteered to participate anonymously from 18 different nationalities (where English was the de facto and de jure language for 10 of them). Eighty-two identified as women and twenty-five identified as men; *M*age = 29.92, *SD* = 7.31, age range: 19–70 years. Participants were asked to reflect on a situation “*when they were around other people (e.g., family, friends or colleagues), where they felt it was important for them to be themselves and openly share their feelings*”. Our intention was to awaken the sense of belongingness in the situation where social support from other people was highlighted.

*Participants in condition 2 (social-value representation):* 94 participants volunteered to participate anonymously from 21 different nationalities (where English was the de facto and de jure language for 6 of them). Fifty-nine identified as women and thirty-five identified as men; *M*age = 32.83, *SD* = 9.72, age range: 18–66 years. Participants were asked to reflect on a situation “*when they were around other people (e.g., family, friends or colleagues), where they felt it was important for them to need to be valued and not be compared to others*”. Our intention was to awaken the sense of belongingness in the situation where personal experience of social value, in contrast to assessment based on comparison with others, was highlighted. After these specific conditions were induced, the identical instruction for both conditions followed: “*Now, think back to the situation you just wrote down, please indicate how you felt during that moment*”. This instruction was followed by measures of different appraisals that were identically used in both conditions. All items were given a response scale ranging from 1 (not at all) to 7 (very much).

### 3.2. Measures

**Need to belong** was measured using 3 items (α = 0.85) adapted from [65]: “I feel the need to belong with others”, “I needed to belong”, and “It was important for me to feel part of others”.

**Appraisals of exclusion** were measured using 5 items (α = 0.93) adapted from [71]: “I can be rejected by others because of what I have done”, “I think I can be isolated from others because of this”, “I felt rejected thinking about what happened”, “I felt alone thinking about what happened”, and “I felt rebuffed thinking about what happened”.

**Appraisals of shame** were measured using 5 items (α = 0.93) adapted from [71]: “I felt disgrace thinking about this”, “I felt ashamed thinking about what I had done”, “I felt humiliated reflecting on this”, “I felt inferior to others reflecting on what happened”, and “I felt vulnerable thinking about what happened”.

**Appraisals of social-worthiness** were measured using 3 items (α = 0.60) adapted from [65]: “I felt that I needed to convince people about myself”, “I felt that I was useless”, and “I would like for people to respect me more”.

**Appraisals of emotional self-expression** were measured using 4 items (α = 0.90) adapted from [65]: “I felt that I could be myself around people”, “I felt that I could show my emotions to others”, “I felt I was allowed to express my emotions around others”, and “I got what I wanted emotionally from others”.

***Appraisals of prosocial-relating behaviour*** were measured using 5 items (α = 0.79) inspired from research reflecting prosocial action in sharing, helping, and comforting [72]: *“I am more patient with other people in that situation”, “I wanted to be more social in that situation”, “The situation made it easier to relate to other people, “I am more likely to be honest in that situation”,* and *“This situation made me more inclusive to other people”*.

## 4. Results

Statistical analyses were performed on both datasets separately using IBM SPSS 28, and descriptive statistics are provided in Table 1 (condition 1) and Table 2 (condition 2) with the dependent variables (need to belong) on the top. Residual and scatter plots indicated that the assumptions of normality, linearity and homoscedasticity were all satisfied [73]. In condition 1 (see Table 1), the need to belong correlated significantly with appraisals of social-worthiness (*r* = 0.35, *p* < 0.01), prosocial-relating behaviour (*r* = 0.33, *p* < 0.01), and emotional self-expression (*r* = 0.28, *p* < 0.01), while correlations with appraisals of exclusion and shame were non-significant. The measures of exclusion, shame and social-worthiness were, as expected, strongly intercorrelated, thus providing support for the convergent validity of the used instruments.

In condition 2 (see Table 2), the need to belong was correlated significantly with all measures in the study. More specifically, the need to belong was significantly correlated with appraisals of exclusion (*r* = 0.36, *p* < 0.001), shame (*r* = 0.41, *p* < 0.001), social-worthiness (*r* = 0.49, *p* < 0.001), prosocial-relating behaviour (*r* = 0.43, *p* < 0.001), and emotional self-expression (*r* = 0.24, *p* < 0.01). Again, moderate to strong correlations between variables in the study were found supporting convergent and discriminant (e.g., negative correlations between social-worthiness and emotional self-expression) validity of the instruments in the study. The overall analysis of the psychometric associations between the study variables clearly showed that the relation between the dependent variable (need to belong) and independent variables (appraisals of exclusion, shame, social-worthiness, prosocial-relating behaviour, and emotional self-expression) was stronger in condition 2 compared to condition 1.

### 4.1. Comparison of Mean Levels in Two Conditions

We performed a series of separate t-tests on all independent variables to detect a potentially consistent pattern regarding the levels of reported experiences. Mean levels and standard deviations are reported in Table 1 and Table 2. Although we found a clear tendency that showed higher mean levels in condition 1, compared to condition 2, this difference was not found to be statistically significant for appraisals of exclusion, shame and social-worthiness. However, the mean levels of the two variables that aimed to measure positive personal processes (i.e., appraisals of emotional self-expression and prosocial-relating behaviour) were both found to be statistically different across conditions 1 and 2 at *p* < 0.01 and *p* < 0.001, respectively. This finding indicates that positive tendencies, here measured as emotional self-expression and prosocial-relating behaviour, might be suppressed in condition 2 “social-value representation” where social-worthiness and comparison processes are accentuated, compared to condition 1 “social-emotional support” where more expressions of true emotions and interpersonal support were central. These tendencies are further elaborated upon in the discussion part of the paper.

### 4.2. Predicting Need to Belong

Considering that descriptive analysis based on intercorrelations between the measures in the study indicated the existence of two different patterns between dependent and independent variables, a stepwise regression approach was used to illuminate these relations. Notably, age and gender were also introduced into the regression on the need to belong as a precautionary role based on a diverse population, in relation to the motivations. The six independent variables in the study were regressed on the need to belong in six distinct steps, in both conditions. Table 3 refers to condition 1, and Table 4 to condition 2. The rationale behind this decision is two-fold. First, the point of departure in the present study was not based on the specific theoretical model that dictates which variable should be included in the analysis, and more importantly in which order. Thus, although we have previously pointed out that there exists a strong theoretical rationale for examining the relationship between the need to belong and listed personal appraisals in different belongingness-conditions, the study nevertheless has an explorative approach based on the relative novelty of the theoretical proposal that the need to belong and belongingness-conditions could be differentiated. Second, a stepwise approach and entering independent variables one after another might reveal the existence of possible interactions between independent variables and dependent variables in terms of mediating effects.

In condition 1 (see Table 3), the six independent variables were able to account for a total of 24% of the variance in the need to belong (adj. *R*^2^ = 0.24, *p* < 0.01). In the first step, the control variables of age and gender were entered and were found to not be significantly (ns) associated with the effect on the need to belong (*β* = 0.10, *p* = 0.30) and (*β* = −0.02, *p* = 0.82). The second step, exclusion was entered as an appraisal and the effect was, in turn significant *(β* = 0.21, *p* = 0.04), as an appraisal. Yet, in the third step, where the effect of appraisal on exclusion and shame was included, it was non-significant (*β* = −0.01, *p* = 0.97) and (*β* = 0.26, *p* = 0.22). However, the inclusion of the appraisals of social-worthiness, prosocial-relating behaviour, and emotional self-expression on the third, fourth, fifth, and sixth steps increased the total explained variance to 24%. The three variables in question were significant predictors of the need to belong. Beta values in the sixth step for social worthiness were (*β* = 0.37, *p* < 0.001), for prosocial-relating behaviour in the fifth step (*β* = 0.30, *p* < 0.001), and for emotional self-expression in the sixth step (*β* = 0.29, *p* = 0.007). Note that prosocial-relating behaviour initially emerged as a significant predictor at step five, but its impact was reduced in step six (*β* = 0.18, *p* = 0.07). According to Baron and Kenny [74], mediation effects are at work when a third (i.e., assumed mediating variable) accounts for a relationship between independent and dependent variables such that the effects of independent variables are significantly reduced when a mediating variable is included in the regression analysis. As noted earlier and evident in Table 3, the reduction of beta values in the sixth step for prosocial-relating behaviour was reduced after emotional self-expression was included. This reduction called for a more specific mediational analysis where three possible paths were modelled: the relationship between the independent variable (prosocial-relating behaviour) and the dependent variable (need to belong) in model 1, the relationship between the independent variable (prosocial-relating behaviour) and the mediator (emotional self-expression) in model 2, and the relationship between mediator (emotional self-expression) and the dependent variable (need to belong) in model 3. However, the z-value based on the Sobel test for explorations of mediational effects was non-significant (Sobel z-value 1.75, *p* = 0.08). Thus, although mediational effects were indicated in the regression analysis, this relation was found to be not significant, probably due to a low number of participants included in belongingness-conditions.

The explanatory strength of the present model was distinctly stronger in condition 2 (see Table 4) where the six independent variables were able to account for nearly double the total of explained variance in the need to belong (adj. *R*^2^ = 0.40), compared to condition 1. In addition, and again in contrast to condition 1 where the effect of appraisals of exclusion and shame were not significant in step three, these variables alone were now able to explain 13% of the variance in the need to belong. However, the considerable reduction of the appraisal of exclusion from significant (*β* = 0.36, *p* < 0.001) in the second step to non-significant in the third step (*β* = 0.01, *p* = 0.96), clearly indicated that shame had a mediating effect on the relationship between exclusion and the need to belong. The more specific analysis of the above-mentioned mediational effects in the form of Sobel test showed significant values, thus indicating shame as a mediator between appraisal of exclusion and the need to belong (Sobel z-value 2.04, *p* = 0.04). Similar to condition 1, the inclusion of the appraisals of social-worthiness, prosocial-relating behaviour, and emotional self-expression on the third, fourth, fifth, and sixth steps increased the total explained variance to 40%. All these three variables were significant predictors of the need to belong. Beta values in the sixth step were (*β* = 0.48, *p* < 0.001) for social-worthiness, (*β* = 0.025 *p* = 0.01) for prosocial-relating behaviour, and (*β* = 0.26, *p* = 0.01) for emotional self-expression.

## 5. Discussion

The experience and the feeling of belongingness convey a serious and fundamental effect on human existence, and closely connected to this are one’s appraisals of positive and negative social bonds and relationships that coexist in a complex manner. Based on the presented theoretical background, the point of departure in the present study was to explore the relationship between the need to belong and a number of personal appraisals under two different belongingness-conditions. The selected appraisals consist of exclusion, shame, social-worthiness, emotional self-expression, and prosocial-relating behaviour. The overall results indicated that the proportion of explained variance in the need to belong was considerably higher in the condition termed “social-value representation” (condition 2), compared to the condition termed “social-emotion support” (condition 1). This finding is not entirely surprising, considering that people have a strong motivation to exercise the way the self is viewed by others and are in favour of not only being accepted in general social norms but also concerned with one’s sense of self-worth [75]. Research supports that one’s sense of belongingness to a group is in relation to an evaluative element, a cognition of acceptance from external influence [76]. Thus, the appraisal of positive and negative self-identification simultaneously impacts one’s social identity within a given context [77]. Therefore, one could state that a failure to “socially self-represent” oneself is indicative of far more concern to people when negative feelings and experiences of social-worthiness are appraised. In other words, due to the prefix gratification and belief that the way people are socially represented should be in accordance with the way people want it to be, and if not, a negative sense of self is felt, based on a self-defect sense of worth to not favourably represent oneself socially [60,78,79].

The effect of appraisal of shame on the need to belong was evident in belongingness-condition 2 “social-value representation”, thus supporting a theoretical argument that when people are socially excluded from one’s need to “socially self-represent” or attribute oneself to the irrespective belief of group membership, the psychological association is lost, and shame is felt. Studies have shown that people may experience shame when the group they socially identified with do not reciprocate similar feedback of acceptance; thus, causing an intrapersonal handicap in one’s sense of attachment, which leads to the bigger picture of a lack of sense of belongingness (e.g., [80,81,82]).

This need to maintain the ideal self (i.e., to socially represent oneself) is a familiar echo in psychological needs theory especially in the motive to construct the self-concept [75,83,84]. Previous research has indicated how self-worth is conditioned by psychological distress when there is a need for people to meet a certain condition and it is beyond their reach (e.g., [85]). Given the importance for people to experience a positive and congruent self-image, this research further emphasised that the relation between the need to belong and the appraisal of social-worthiness is potent when one’s self-actualising tendency to socially self-represent as a belongingness-condition is denied. Moreover, [65] highlighted that people have a need to be valued and accepted for who they are by others, and at the same time to not be compared with others. Past studies have shown that when one’s agency to self-represent is thwarted, such negative experiences such as being excluded from social bonds stimulates a more anxious feeling in people [27,86] and life satisfaction is reduced alongside the sense of significance (e.g., [24,87,88]). Furthermore, studies have also demonstrated that a negative sense of worth, leads to a range of negative emotions, which include *shame* (e.g., [27]). As previously mentioned, the rationale behind the appraisal of social-worthiness was its relation to the feeling of shame on a theoretical level [45]. For example, this motivation to maintain a positive social image of oneself. As such, similarly, according to Brown [58], an inconsistent and damaged sense of connection and belongingness subjects people to feeling shame based on this need to fit into one’s sense of social image.

The results of this study have indicated the link between social-worthiness and shame in the way that it may be conceptualised and assessed, as both a stable and temporary construct. For example, how the appraisal of exclusion became trivial when shame was introduced as a second appraisal. However, overall, both exclusion and shame became extraneous when social-worthiness was appraised. This entry suggests that, while a person’s sense of belongingness can be relatively stable over time, it can also fluctuate depending on the belongingness-conditions and the appraisals attached to it. One’s appraisal of social-worthiness is important and consequential for individuals in society owing to the perceived individual’s comparison and relationship with others. Critically, evidence has shown that people use status-based understanding and experience to regulate their emotions and behaviour (e.g., [89]). As indicated in this study, having (high or low) social-worthiness is associated with one’s sense of belongingness and as such, this appraisal is sufficient to cause a swing in the evaluation of the need to belong. Moreover, even when objective manipulation is compared, e.g., prosocial-relating behaviour in relation to one’s sense of self-interest in gaining belongingness [72]. Social-worthiness still has a stronger impact, and this could be because of how we as humans have come to live, i.e., this evolutionary importance of knowing one’s social reference in a given society as such, being valued and accepted for oneself while not being compared to others and fulfilling this need to belong (e.g., [2,89]).

Relatedly, constructs such as social comparison, social status, self-esteem, and self-regard are more relative, quicker, and convenient for perceiving one’s self-evaluation and interest for a sense of belongingness. As indicated, the emotional and psychological pull of these constructs is powerful (e.g., [57,90,91,92]). In sum, the interest in social-worthiness in reference to the need to belong and a sense of belongingness plays a better role in comparison to the appraisals of exclusion, shame, emotional self-expression, and prosocial-relating behaviour. While this is the overall finding, ultimately it is critical to be aware of the individual difference variables and belongingness-condition factors that can influence the extent or direction of the impact of sense of belongingness on the need to belong. Thus, although it seems obvious in one way that social-worthiness is an important component of the self, it is less clear what the precise determining factors are, as it not only reflects the perception of one’s sense of belongingness, yet, on the other hand, it reflects the perception of one’s sense of worth or regard from others, as indicated in the result when positive tendencies, e.g., emotional self-expression and prosocial-relating behaviour became trivial when social-worthiness was present.

## 6. Limitations and Implications

As in any research study, there exist several potential limitations that should be addressed. First, the limitation that needs to be addressed is that the data were collected during the coronavirus disease (COVID-19) global pandemic. Thus, it is important to be open about how this period impacted the participants and indirectly influenced the results. Second, while we explored the notion of belongingness, no specific measure of different attachment styles was used, which may impact the effect and sensitivity of the variables. Third, it is also important to consider how people of diverse cultures may impact the interpersonal nature of belongingness conditions. As such, one might say that we failed to consider this as we did not have any measurement of the cultural effect. For example, between Asian and Western, the impact of culture can be less clear when it comes to the need for “social-emotional support” and “social-value representation”. Fourth, we did not examine a larger constellation of constructs that can be used to measure a person’s sense of evaluation (e.g., self-esteem). Future research could incorporate such constructs towards further understanding. Moreover, important factors remain to be investigated, for example, how group norms or the multifaceted nature of different social contexts affect the fundamental need to belong.

Notwithstanding these limitations, the present study offers several theoretical and empirical implications. This is, to our knowledge, the first study that explicitly explores the need to belong under different belongingness conditions. Thus, the study contributes to existing knowledge of how the relationship between the need to belong and the appraisal of social worthiness is effective as a need to socially self-represent for belongingness. If social exclusion is decided, in part, by one’s emotional appraisal of sense of belongingness, then different belongingness conditions can explicitly influence individuals’ motivation as well as interpersonal interactions. The present study highlights and contributes nuancing concepts that are traditionally used interchangeably, e.g., the need to belong and the sense of belongingness. Therefore, as both targets and source (belongingness and social exclusion), the type of belongingness conditions may impact how the form and experience of both sense of belongingness and social exclusion are addressed.

## 7. Conclusions

Conceptualised broadly as a fundamental human need, the present review paints a complex picture in which people navigate the “hold” between belongingness and social exclusion. As in countless situations, people have limited insight into each other’s needs and feelings. Moreover, people have different levels of needing to belong, some are stronger than others based on their social condition and interactions. This study reflects on two different belongingness-conditions: “social-emotional support” and “social-value representation” and further nuance how different conditions might affect the fundamental need to belong. Although both belongingness-conditions are of importance as a motivational need, this study shows that the need to belong can be accentuated by a belongingness that is materialised by satisfying one’s sense of worth as appraised by social-worthiness. While belongingness is often characterised as a categorically positive condition juxtaposed to “not-belonging”, it is encouraging to evaluate the way we socially engage and identify with the world, as there are stark differences between the conflicting ways people can socially represent and interpret a sense of belongingness, for example, between self-identifying, self-categorising, self-stereotyping, and fitting in, from the appraisal of shame.

## Figures and Tables

**Table 1:** Correlations and descriptive statistics among study variables in condition 1.

Variables	1	2	3	4	5	6
1. Need to belong	1	0.17	0.19	0.35 **	0.33 **	0.28 **
2. Exclusion		1	0.87 ***	0.61 ***	0.14	−0.32 **
3. Shame			1	0.59 ***	0.12	−0.20 *
4. Social-worthiness				1	0.10	−0.15
5. Prosocial-re bhv					1	0.39 ***
6. Em self-expression						1
MEAN	5.01	3.02	2.87	3.60	5.29	4.82
SD	1.46	1.65	1.61	1.39	1.28	1.42

* *p* < 0.05; ** *p* < 0.01; *** *p* < 0.001.

**Table 2:** Correlations and descriptive statistics among study variables in condition 2.

Variables	1	2	3	4	5	6
1. Need to belong	1	0.36 ***	0.41 ***	0.49 ***	0.43 ***	0.24 **
2. Exclusion		1	0.87 ***	0.66 ***	0.12 *	−0.21 *
3. Shame			1	0.60 ***	0.13	−0.09
4. Social-worthiness				1	0.14	−0.28 **
5. Prosocial-re bhv					1	0.40 ***
6. Em self-expression						1
MEAN	4.64	2.96	2.66	3.90	4.76	4.40
SD	1.68	1.80	1.68	1.55	1.54	1.52

* *p* < 0.05; ** *p* < 0.01; *** *p* < 0.001.

**Table 3:** Regressing need to belong on age and gender (step 1) exclusion (step 2), shame (step 3), social-worthiness (step 4), emotional self-expression (step 5), and prosocial-relating behaviour (step 6) in condition 1.

Steps	Predictors Entered	Adj. *R^2^*	F_change_	β
1	Age and gender	−0.01	0.54	0.10/−0.02
2	Exclusion	0.02	4.35 **	0.21
3	Exclusion	0.03	1.51	−0.01
	Shame			0.26
4	Exclusion	0.11	10.09 ***	−0.17
	Shame			0.16
	Social-worthiness			0.38 ***
5	Exclusion			−0.22
	Shame			0.16
	Social-worthiness	0.19	11.74 ***	0.38 ***
	Prosocial-relating behaviour			0.30 ***
6	Exclusion	0.24	7.60 **	−0.01
	Shame			0.08
	Social-worthiness			0.37 ***
	Prosocial-relating behaviour			0.18
	Emotional self-expression			0.29 **

** *p* < 0.01; *** *p* < 0.001.

**Table 4:** Regressing need to belong on age and gender (step 1) exclusion (step 2), shame (step 3), social-worthiness (step 4), emotional self-expression (step 5), and prosocial-relating behaviour (step 6) in condition 2.

Steps	Predictors Entered	Adj. *R^2^*	F_change_	β
1	Age and gender	−0.01	0.40	−0.08/−0.05
2	Exclusion	0.11	12.13 ***	0.36 ***
3	Exclusion	0.13	4.16 *	0.01
	Shame			0.41 ***
4	Exclusion	0.23	12.75 ***	−0.23
	Shame			0.35 **
	Social-worthiness			0.43 ***
5	Exclusion			−0.21
	Shame			0.32 *
	Social-worthiness	0.36	18.32 ***	0.39 ***
	Prosocial-relating behaviour			0.37 ***
6	Exclusion	0.40	7.15 **	−0.11
	Shame			0.21
	Social-worthiness			0.48 ***
	Prosocial-relating behaviour			0.25 *
	Emotional self-expression			0.26 **

* p < 0.05; ** *p* < 0.01; *** *p* < 0.001.

## Data Availability

The raw data supporting the conclusions of this article will be made available by authors, without undue reservation.
